# Soil-transmitted helminths and schistosome infections in Ethiopia: a systematic review of progress in their control over the past 20 years

**DOI:** 10.1186/s13071-021-04600-0

**Published:** 2021-02-05

**Authors:** Rosie Maddren, Anna Phillips, Alison Ower, Toby Landeryou, Birhan Mengistu, Ufaysa Anjulo, Ewnetu Firdawek, Nebiyu Negussu, Roy Anderson

**Affiliations:** 1 London Centre for Neglected Tropical Disease Research (LCNTDR), Department of Infectious Disease Epidemiology, School of Public Health, Faculty of Medicine, Imperial College London, London, UK; 2 Children Investment Fund Foundation, London, UK; 3 Disease Prevention and Health Promotion Core Process, Ministry of Health, Wolaita, Ethiopia; 4 Bacterial, Parasitic and Zoonotic Diseases Research Directorate, Ethiopian Public Health Institute, Addis Ababa, Ethiopia; 5 Neglected Tropical Diseases, Federal Ministry of Health, Addis Ababa, Ethiopia

**Keywords:** Soil-transmitted helminths, Schistosomiasis, Transmission break, Ethiopia, Control programme, Prevalence, Intensity, Age-prevalence relationship, Mass drug administration, The 2030 Neglected tropical disease roadmap

## Abstract

**Background:**

Ethiopia has set the ambitious national targets of eliminating soil-transmitted helminths (STH) and schistosomiasis (SCH) as public health problems by 2020, and breaking their transmission by 2025. This systematic review was performed to provide insight into the progress made by the national STH and SCH control programme purposed with reaching these targets.

**Methods:**

Studies published on STH and SCH in Ethiopia were searched for using Web of Science, PubMed, Scopus, and the resulting references of selected studies. Prevalence and intensity were analysed, stratified by region, age, and diagnostics.

**Results:**

A total of 231 papers published between 2000 and 2020 were included. Over the past two decades, *Trichuris trichiura* (TT) infection has shown the most statistically significant decrease (93%, *p* < 0.0001), followed by *Schistosoma mansoni* (SM) (69%, *p* < 0.0001), *Ascaris lumbricoides* (AL) (67%, *p* < 0.0001) and *Schistosoma haematobium* (83%, *p* = 0.038) infections. Geographically, parasite burden has only consistently shown a significant reduction in the Southern Nations, Nationalities and Peoples’ Region of Ethiopia, where AL, TT, hookworm and SM significantly decreased by 80% (*p* = 0.006), 95% (*p* = 0.005), 98% (*p* = 0.009) and 87% (*p* = 0.031), respectively. Prevalence of STH was highest among adults across all species, contrary to typical age-infection profiles for TT and AL that peak among school-aged children. Expanding treatment to the whole community would target reservoirs of adult and preschool-aged infection within the community, assisting Ethiopia in reaching their national transmission break targets. There was substantial heterogeneity in diagnostic methods used across studies, the majority of which predominantly used single-slide Kato–Katz. This low slide frequency provides poor diagnostic sensitivity, particularly in low endemic settings.

**Conclusion:**

The prevalence of STH and SCH in Ethiopia has decreased over time due to the strategic use of anthelmintics. Both standardising and increasing the sensitivity of the diagnostics used, alongside the ubiquitous use of parasite intensity with prevalence, would enable a more accurate and comparable understanding of Ethiopia’s epidemiological progress. Further work is needed on community-wide surveillance in order to understand the burden and subsequent need for treatment among those outside of the standard school-based control program.

**Graphical Abstract:**

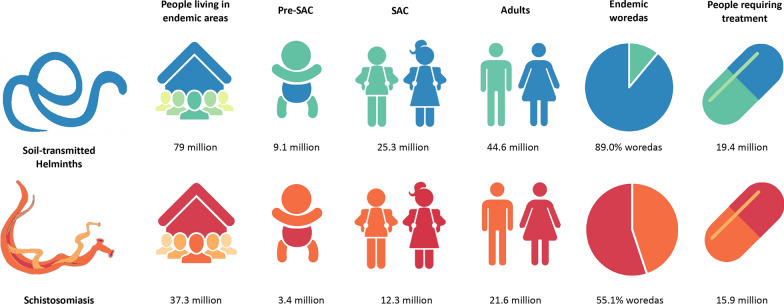

## Background

Soil-transmitted helminths (STH) and schistosomiasis (SCH) are the most widespread neglected tropical diseases (NTDs) globally [1]. Globally, 1.5 billion and 240 million individuals are estimated to be infected with STH and SCH, respectively [2, 3]. In Ethiopia, 36 million and 5 million individuals are infected with STH and SCH, respectively, often in remote and impoverished areas [4, 5]. Recent analysis has estimated Ethiopia to have the 13th highest prevalence of both STH and SCH among over 40 countries in Africa [6].

STH is a collective term for roundworm *(Ascaris lumbricoides*; AL), whipworm *(Trichuris trichiura*; TT), hookworm (*Necator americanus*, *Ancylostoma duodenale* and *Ancylostoma ceylanicum*; HW), and threadworm (*Strongyloides stercoralis*; SS). STH eggs excreted in infected individuals’ faeces contaminate soil and water sources, and thus their prevalence can be considered a proxy marker for poor sanitation and hygiene. AL and TT infections are transmitted via the faecal-oral pathway where individuals become infected after ingesting eggs in water or from fomites, hands, soil, or food. HW and SS infections are propagated transdermally by infective stages penetrating skin in contact with soil. These distinct transmission pathways impact the age-infection profile of infection.

The blood flukes of the genus *Schistosoma* cause either urogenital (*Schistosoma haematobium*; SH) or intestinal (*Schistosoma mansoni*; SM) SCH. Eggs are excreted in a species-dependent manner, either in host faeces (SM) or urine (SH). SM and SH continue their life cycles from the egg stage via aquatic snail intermediaries from which they ultimately re-infect human hosts by penetrating skin in contact with infested water bodies.

STH and SCH infections can both be controlled through the periodic administration of preventive chemotherapy (PC) with albendazole (ALB) and praziquantel (PZQ), respectively. Treatment is largely conducted through the national STH and SCH control program, where PZQ distribution to school-aged children (SAC) for SCH is combined with ALB for STH in co-endemic regions [7]. Serendipitously for STH control, 69 districts are co-endemic for lymphatic filariasis whereby whole communities (pre-SAC, SAC and adults) will receive ALB for LF, which will have a beneficial impact on STH control. Where LF and SCH are non-endemic, STH is untreated aside from selective deworming of pre-SAC (2–4 years) carried out by health extension workers [8]. Despite the focus of STH control on SAC, several studies have shown significant heterogeneity in exposure to infection by host age. Age-prevalence profiles are typically concave for AL and TT, with children disproportionally burdened with higher prevalence. An age-dependent decline in prevalence is often observed in adults, which is probably due to reduced exposure and age-acquired immunity (see Additional file 1: Figure S1) [9]. Conversely, burdens of HW and SS infection tend to be higher in adults, and are thought to increase with age-related exposure risk such as agriculture-based manual labour [10]. The SAC-focused treatment strategy for STH therefore favours the treatment of AL and TT over that of HW.

Exposure to SCH infection is positively correlated with age, peaking among children aged 9–12 years, as infection risk behaviour changes from passive (mothers bathing infants in infested water) to active (children playing or collecting water in infested water bodies) [11]. As a result, current SCH control relies on mass drug administration (MDA) and monitoring infections in SAC. Pre-SAC are currently not included in any MDA and at-risk adults are included only if the prevalence of SAC is > 50%.

Worm burden within the human population is highly aggregated; a small number of individuals exhibit high worm burdens. High-burden individuals tend to be predisposed to both STH and SCH infections, and exhibit the greatest morbidity [12, 13]. STH and SCH helminths are dioecious species that propagate through sexual reproduction. In order to disrupt transmission, community worm burden must be low enough whereby the chance of both genders at appropriate maturity stages co-habiting the host is improbable. This phenomenon is termed the transmission breakpoint. Defining the prevalence and mean intensity of infection that marks a transmission breakpoint has been a focus of recent work for both STH and SCH [9, 14]. It has been suggested that STH infection measured at a prevalence of 2% [verified by quantitative polymerase chain reaction (qPCR)] 24 months after MDA cessation can predict transmission interruption [15–17].

### Epidemiology of STH and SCH in Ethiopia

**Fig. 1:**
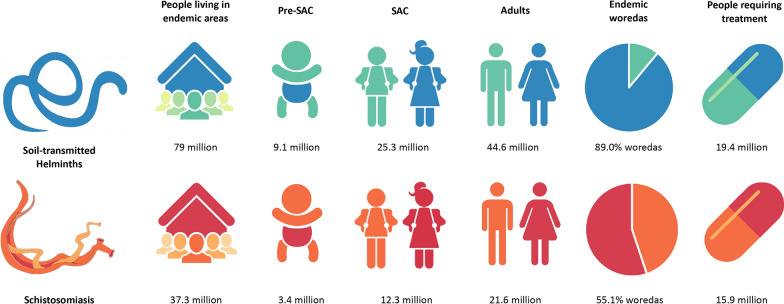
Ethiopian soil-transmitted helminths (STH) and schistosomiasis (SCH) endemicity figures. STH and SCH prevalence in Ethiopia by age group, number of endemic woreda (districts) and people requiring treatment according to data published in 2017 [4] Illustration drawn by the authors

National mapping surveys conducted between 2013 and 2014 suggested widespread STH infection, distinct regional distribution of SM infection, and SH infection focalised on the Rift Valley region [4, 18]. High STH infection prevalences are found in predominantly western regions of Gambella, Oromia and northern Southern Nations, Nationalities and Peoples’ Region (SNNPR). SCH was found at high prevalence in northerly regions of Amhara, Tigray, Benishangul-Gumuz and Gambella. More specifically, mapping found 476 and 346 districts to be endemic for STH and SCH, respectively, with 229 districts co-endemic for STH/SCH. The endemicity risk map of STH and SCH is shown in Additional file 2: Figure S2 and graphically represented in Fig. 1 [4].

### STH and SCH control in Ethiopia

Over the past decade, Ethiopia has made great efforts toward STH and SCH control through multiple rounds of mass PC in endemic areas. The Enhanced Outreach Strategy was launched in 2004, for the deworming of pre-SAC biannually in every district alongside vitamin A distribution, and achieved 77% coverage in 2017 [19]. National STH and SCH deworming of SAC was initiated in 2007, with the treatment of 1 million SAC for STH and SCH [7]. A further 11.6 million and 7.8 million SAC were treated in 2013 and 2014 for STH, respectively, with 1.07 million SAC treated for SCH in 2013. National STH and SCH mapping (Additional file 2: Figure S2) was completed in 2013 [4, 18]. The national STH and SCH control programme that was launched in 2015 was designed to achieve the elimination of STH and SCH as major public health problems by 2020, and to reach transmission break by 2025 [4]. By the end of 2015, thirteen million and 7.9 million SAC had been treated for STH and SCH, respectively [7]. Adolescents (15–19 years old) have been treated for STH in 86 priority woredas (districts) since 2015 [20].

The national LF elimination programme was launched in 2009 exclusively in Gambella, but had expanded into woredas in SNNPR, Oromia, Amhara, and Benishangul-Gumuz by 2015, whereby 3.7 million individuals were treated community-wide with ALB [7, 21]. Co-infection with LF was reported in the woredas where STH were found at the highest prevalences, thus these woredas benefitted from the additional treatment of older age groups with ALB for LF (Additional file 3: Figure S3) [22].

### World Health Organisation 2030 targets

In the recently released 2030 roadmap, the World Health Organisation (WHO) has set a global target to “achieve and maintain elimination of STH morbidity in pre-SAC and SAC by 2030” by reducing the “number of countries where prevalence of pre-SAC and SAC with STH infections of moderate and heavy intensity less than 2%” [23]. SCH control guidelines have yet to be released, but the WHO aims to “eliminate SCH as a public health problem” by reducing the “proportion of heavy intensity SCH infections to less than 1%” [24]. The present paper reviews epidemiological studies of STH and SCH in Ethiopia published between 2000 and 2020, with a view to highlighting the achievements made so far over the past two decades, and how the continuation of control efforts will aid towards Ethiopia’s national target of reaching transmission break by 2025 and furthermore reaching the new WHO 2030 targets.

### Methods

This systematic review was completed in accordance to the preferred reporting items for systematic reviews and meta-analyses (PRISMA) guidelines (see Additional file 4: Figure S4). Electronic searches of the Web of Science (encompassing Medline, Embase, and Global Health Databases), PubMed and Scopus were carried out for articles published from 2000 to August 2020 in English, using search terms “(geohelminth* OR helminth* OR *Ascaris* OR *lumbricoides* OR *Ascariasis* OR *Trichuris* OR *trichiura* OR Trichuriasis OR Hookworm OR *Necator* OR *americanus* OR *Ancylostoma* OR *duodenale* OR *Schistosoma* OR Schistosoma* OR *mansoni* OR *haematobium* OR *haematobium* OR Schistosomiasis OR Bilharzia OR roundworm OR whipworm OS *strongyloides* OR *stercoralis* OR threadworm OR nematode*) NOT (dog OR goat OR canine OR ruminant OR camel OR non-human primate OR fish) AND (Ethiopia OR Afar OR Amhara OR Benishangul-Gumuz OR Gambela OR Harari OR Oromia OR Somali OR "Southern Nations, Nationalities, and Peoples’ Region" OR SNNPR OR Tigray)”. These searches were complemented with searches in general search engines (e.g. Google Scholar). Finally, the bibliographies of relevant papers identified for inclusion were also searched to identify additional relevant publications. Selected papers were exported to Mendeley (Elsevier, London, UK), and extracted data were analysed using R version 3.6.1.

Articles were initially screened by abstract content, and those that did not investigate either prevalence or intensity in at least one of the six STH or SCH infections were discarded. Full-text screening included studies if they were observational (cross-sectional, prospective cohort, case–control, or retrospective cohort) or were a controlled clinical trial which reported the baseline prevalence or incidence of STH or SCH. Studies of individual case reports, sensitivity and specificity diagnostic comparisons, co-infection investigations, and research on distinct populations, predominantly pregnant women, food handlers and prisoners, were excluded (Additional file 4: Figure S4).

Prevalence and intensity analyses were conducted using the date of data collection and not publication, due to the time lag between the two. Thus, this review includes data collected between 1994 and 2019. Three publications omitting to report a study date were assumed to have been conducted 1 year prior to the publication date. Studies occurring over several years were recorded by year of baseline data collection. When the calculation of mean intensity was not specified, the arithmetic mean was assumed to have been employed. When no age range of participant age category was detailed, the following assumptions were made: pre-SAC (0–4 years), SAC (5–18 years), adults (19–80 years) and community (1–80 years) [25]. Where Global Positioning System coordinates were not provided, the school or community were identified manually using Google Maps. If multiple locations or years were studied, the data for each year or location were recorded discretely if they each fulfilled the data-extraction criteria given above. There were ten instances of papers containing multiple unique datasets, generating 36 additional data points.

Pearson’s correlation coefficient (*r*) was used to measure the linear correlation of parasite prevalence and intensity over time on a national and/or regional scale. Pearson’s correlation was employed to measure the association, if any, between the two continuous variables providing the magnitude and direction of correlation. Statistical significance was set at *p*-values less than 0.05.

## Results

### Overview of selected papers

From the systematically identified 1101 publications, 231 were accepted against the above criteria (Additional file 5: Table S1). Ten studies provided an additional 36 datasets, as previously explained, bringing the final total to 267 datasets to form the basis of the analysis for this review. The publication date, study initiation date and duration, study population, study type, number of participants, gender, age range and mean, STH and SCH prevalence and intensity, diagnostic employed, chemotherapeutic treatment given, region, woreda, kebele and Global Positioning System coordinates of the study sites were extracted for analysis.

Of the 267 datasets meeting the review criteria, STH and SCH prevalence were reported for all nine Ethiopian regions. The majority of studies were implemented in Amhara (38%), Oromia (25%), and SNNPR (22%) (Additional file 6: Figure S6). Sample population focus has shifted over the last 20 years, moving from a community-wide perspective, to focus on SAC and pre-SAC. Overall, 63% studies in this review sampled SAC, with 9% and 28% studies using pre-SAC and community-wide samples, respectively.

Most studies were cross-sectional by age and gender (82%), with sample sizes varying between 55 and 62,520 participants (mean 1506 participants). Participant gender was generally equal between females (mean 49%, 11–68%) and males (mean 51%, 32–89%). A total of 402,189 stool samples were analysed. The proportion of these 267 datasets reporting on AL, TT, HW, SS, SM, and SH infections were 24%, 22%, 21%, 9%, 22%, and 2%, respectively. There was a lack of publications found on SS and SH in particular.

Table 1 gives an overview of the contents of the 231 papers examined in this review and the subsequent 267 unique datasets (for full details see Additional file 5: Table S1), displayed for each parasite.

**Table 1:** Breakdown of the selected 231 papers. The number of papers reporting the diagnostic, treatment, study population and epidemiological parameters are shown for each parasite species

Study element		AL	TT	HW	SS	SM	SH
Parasite studied^a^ (*n* = 267)		24% (*n* = 213)	22% (*n* = 194)	21% (*n* = 197)	9% (*n* = 78)	22% (*n* = 198)	2% (*n* = 21)
Diagnostic	KK and/or FECT	107	101	95	24	125	15
	Other^b^	70	59	68	35	45	5
	Multiple^b^	21	19	19	9	13	0
	Unspecified	15	15	15	10	15	1
Treatment	PZQ/ALB/MBZ	46	44	38	8	59	7
	Not specified	117	106	112	48	97	14
	Not mentioned	50	44	47	22	42	0
Study population	Pre-SAC	19	16	15	3	9	0
	SAC	128	118	120	37	120	18
	Community	66	60	62	38	69	3
Epidemiological parameter	Prevalence	213	194	197	78	198	21
	Intensity	46	44	41	–	70	–

*AL Ascaris lumbricoides*, *TT Trichuris trichiura*,* HW* hookworm, *SS Strongyloides stercoralis*, *SM Schistosoma mansoni*,* SH Schistosoma haematobium*,* KK* Kato–Katz,* FECT* formal ether concentration technique,* PZQ* praziquantel, *ALB* albendazole,* MBZ* mebendazole,* Pre-SAC* pre-school-aged children

^a^Percentage of papers that studied each parasite out of the 267 datasets

^b^Includes direct microscopy, McMaster, molecular techniques [polymerase chain reaction (PCR), quantitative PCR], FLOTAC and Mini-FLOTAC techniques, centrifugation, sedimentation and urine diagnostics

### Overall change in prevalence

**Fig. 2:**
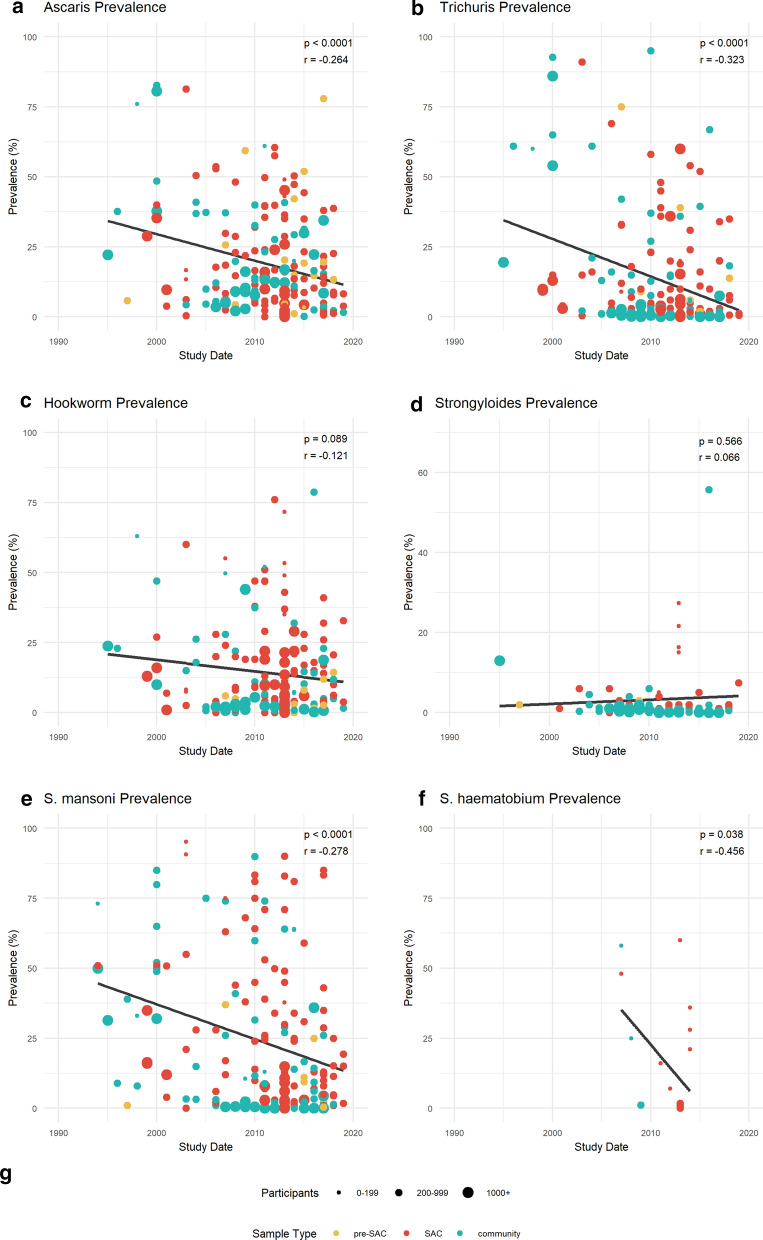
Change in prevalence of STH and SCH between 1994 and 2019. Prevalence of *Ascaris lumbricoides* (AL; **a**), *Trichuris trichiura* (TT; **b**), hookworm (HW; **c**), *Strongyloides stercoralis* (SS; **d**), *Schistosoma mansoni* (SM; **e**), and *Schistosoma haematobium* (SH; **f**) infections between 1994 and 2019. Pearson’s correlation coefficient (*r*;* top right corner* of each plot with associated* p*-value) was used to measure the linear correlation between parasite prevalence and study date. Study populations, differentiating between pre-school-aged children (pre-SAC), SAC and community-wide study populations, are indicated by* different colours*, whilst* point size* indicates study population size (see legend) (**g**). The* trend line* was not weighted by sample size. Note the SS graph* y*-axis range is from 0 to 70, and differs from that of the other plots. For other abbreviations, see Fig. 1

Stratifying each of the 267 datasets by study population, as shown in Fig. 2, shows the decrease in parasite prevalence recorded across the 267 datasets between 1994 and 2019. TT (34–2%, *p* < 0.0001) prevalence shows the most statistically significant decrease over time, with a significant reduction also seen in SM (45–14%, *p* < 0.0001), AL (34–11%, *p* < 0.0001) and SH (35–6%, *p* = 0.038). HW (21–11%, *p* = 0.089) and SS (2–4%, *p* = 0.566) showed a non-significant decrease and increase in prevalence over time, respectively. The increased prevalence shown in SS may be influenced by a single community study (*n* = 792) in 2016 that reported an overall prevalence of 56%. The small sample dataset size for SH (*n* = 21) limits a conclusive trend. The decrease in TT and AL prevalence is seen more convincingly when the trend is assessed for community-wide studies alone, compared to SAC trends. The high level of stochasticity of points in SM and TT plots should be noted.

### Change in prevalence by region

**Fig. 3:**
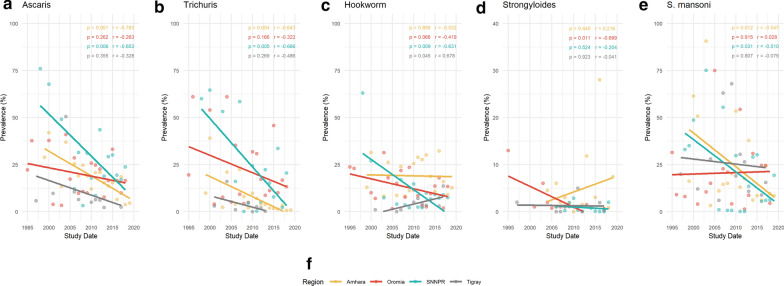
**a**–**e** Regional prevalence change in Amhara (*yellow*), Oromia (*red*), Southern Nations, Nationalities and Peoples’ Region (*SNNPR*; *turquoise*) and Tigray (*grey*). Prevalence change of AL (**a**), TT (**b**), HW (**c**), SS (**d**) and SM (**e**) infections between 1994 and 2019, stratified by region for Amhara (*n* = 102), Oromia (*n* = 67), SNNPR (*n* = 59) and Tigray (*n* = 21). Pearson’s correlation coefficient (*top right corner* of each plot with associated* p*-value) was used to measure the linear correlation over time between parasite prevalence and study date. For other abbreviations, see Fig. 2

Parasite data were available for each of the nine regions of Ethiopia, and were sufficient to stratify prevalence in Amhara (*n* = 102), Oromia (*n* = 67), SNNPR (*n* = 59) and Tigray (*n* = 21) over time. Figure 3 highlights regional successes and those regions that require further attention. The greatest parasite reduction is seen in Amhara, where AL (Fig. 3a) reduced by 80% from 33 to 7% (*p* < 0.001). TT and SM (Fig. 3b, e) also significantly decreased in Amhara, by 99% from 19 to 0.1% (*p* = 0.004) and by 83% from 44 to 7% (*p* = 0.012), respectively. SNNPR showed a consistent reduction across all parasite infections compared to other regions, as AL (56-11%, *p* = 0.006), TT (54-3%, *p* = 0.005), HW (31-0.5%, *p* = 0.009) and SM (42-6%, *p* = 0.031) all display significant declines in prevalence. A significant increase in prevalence was seen in Tigray, as HW increased from 0.1 to 7.6% (*p* = 0.045), whilst SS in Amhara and SM in Oromia increased non-significantly.

### Change in prevalence by age

**Fig. 4a–f:**
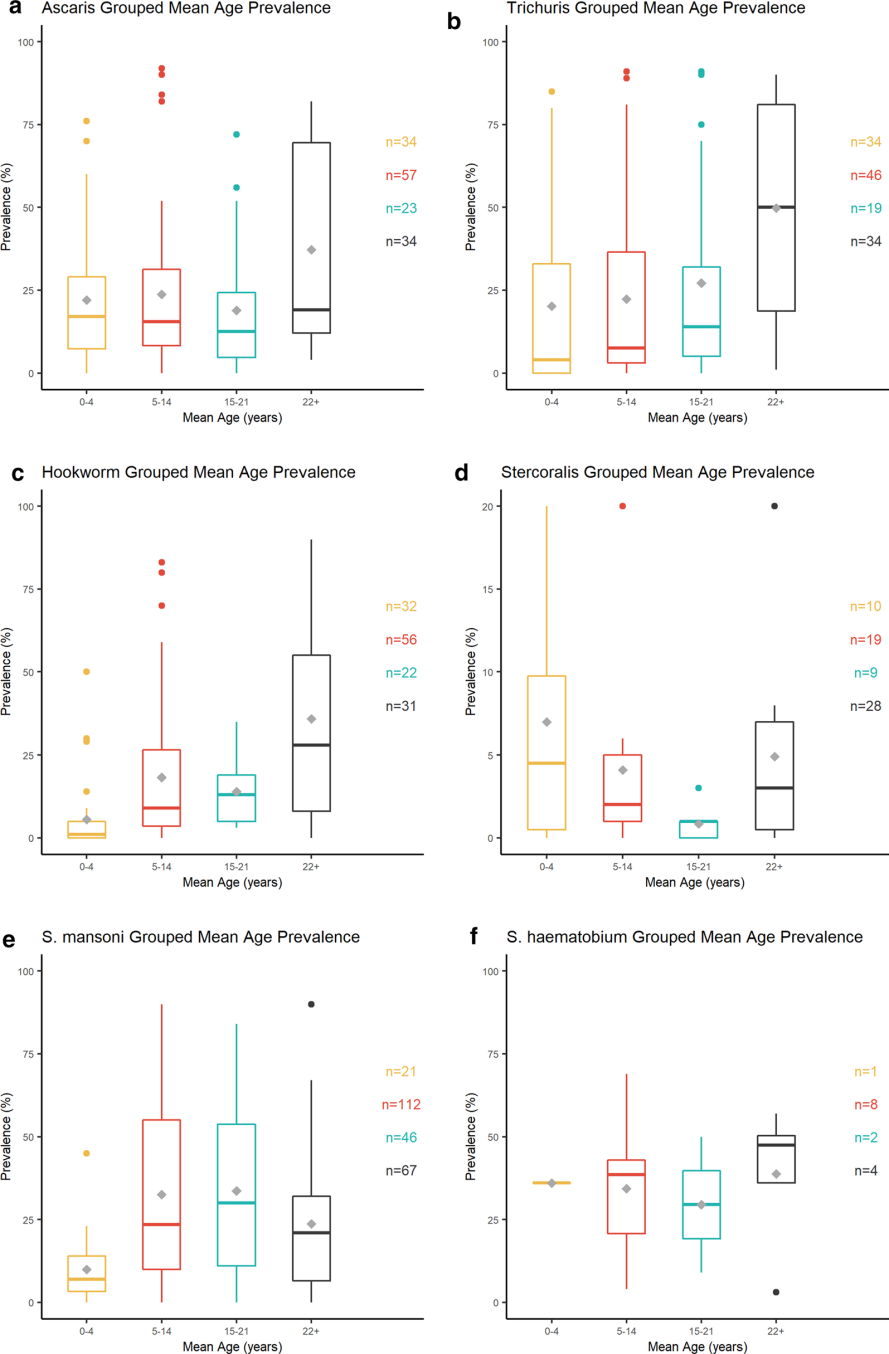
Age prevalence profile. Prevalence of AL (**a**), TT (**b**), HW (**c**), SS (**d**), SM (**e**), and SH (**f**) infections plotted against age group mid-point mean.* Lower boundary* First (25%) quartile, *upper boundary* third (75%) quartile, *inner horizontal line* median (50%). The arithmetic mean of each age group is depicted as a* grey diamond*.* n* Number of studies used to generate each box (*right-hand side* of each plot). Prevalence figures higher than the third (75%) quartile are shown as* individual points*. For abbreviations, see Fig. 2

Prevalence was stratified by age in 109 studies, providing 394 data points for age analysis. Due to the discordant age bands reported between papers, the mid-point means of the age-groupings were calculated and plotted against parasite prevalence (Fig. 4).

Fig. 4 shows a change from the typical age-prevalence profile (Additional file 1: Figure S1) for all species. AL and TT (Fig. 4a, b) showed the most pronounced deviation from the typical distribution of infection, as higher parasite burdens were seen in adults compared to pre-SAC, SAC and adolescents. This is likely a reflection of the historical SAC-focused MDA. HW (Fig. 4c) showed higher than expected infection levels in SAC, yet prevalence remained typically high in adults.

Conversely, SM (Fig. 4e) age prevalence has not differed from the typical age-prevalence distribution as SAC and adolescents are shown to still harbour the majority of infections. Despite the overall reduction in SM recorded for Ethiopia (45-14%, *p* < 0.0001; Fig. 2e), the national (STH and) SCH control program should ensure that high treatment coverage and compliance are maintained to reduce the burden in children.

### Prevalence versus intensity measurements

Consistently throughout the reviewed literature, intensity was reported as a secondary measure to prevalence (Table 1). Of the 267 datasets included in this review, the intensity of infection by egg counts in faecal samples was provided alongside prevalence for 21% of AL (45/212), 22% of TT (43/194), 20% of HW (40/197) and 36% of SM (70/198) studies. These intensity measures included the arithmetic and geometric means, and a tiered scale of light, moderate and heavy intensities based on the published ranges of eggs per gram (epg) counts specified by WHO [26]. A further four papers used other statistics such as median egg counts, or eggs per millilitre of urine for SH. The lack of standardisation of an already sparsely used measure reduces the data available for analysis.

There were sufficient intensity data reported for AL (*n* = 46), TT (*n* = 44), HW (*n* = 41) and SM (*n* = 70) (Additional file 7: Figure S6). Across all four species, a non-significant change in intensity was shown. AL and TT showed a non-significant decrease in epg recorded over time, whilst HW showed a non-significant increase in epg over time. SM showed negligible change over time, fluctuating around a mean of 157 epg. The heterogeneity in AL and TT epg, ranging from 1 to 15,263 epg and 1–3295, respectively, should be noted. Across all species, the highest epg values were recorded in SAC study populations.

The majority of the studies in this review (45%) reported low STH and SCH prevalences. However, due to the non-linear relationship between prevalence and average intensity of infection, whereby prevalence changes negligibly at high intensities and dramatically at lower intensities, the use of prevalence as the predominant epidemiological parameter should be re-examined, especially in low prevalence settings.

**Fig. 5a–d:**
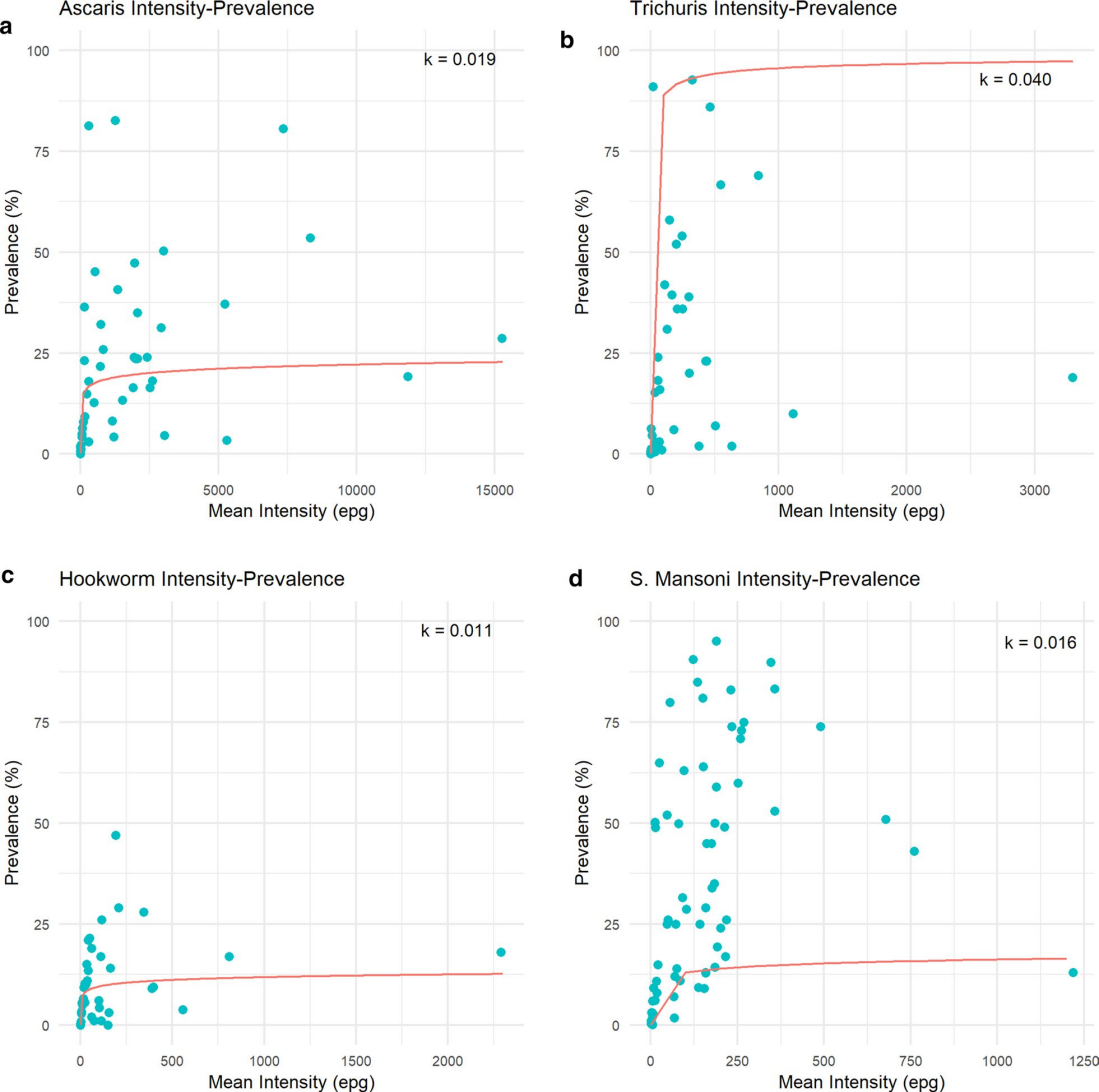
Reported prevalence and intensity. The relationship between reported prevalence and arithmetic intensity for AL (**a**), TT (**b**), HW (**c**), SM (**d**). The negative binomial model is shown fitted for the arithmetic mean, and the estimated negative binomial parameter (aggregation parameter;* k*) value is shown on the* upper right-hand side* of each plot. Note all plots show a range of prevalence of 0 to 100%, whilst the eggs per gram (*epg*) range varies for each parasite. For other abbreviations, see Figs. 2

Figure 5 displays the average reported prevalence plotted against the arithmetic mean intensity of infection, using data points averaged across age classes. The relationship between the two epidemiological parameters is defined by the magnitude of the negative binomial parameter (aggregation parameter; *k*) [27]. Parasites are aggregated in their distributions within the human host population, where the negative binomial probability is typically a good descriptor of the observed data (either egg counts or worm expulsion counts for STH [27]). The relationship is non-linear, where prevalence (*P*) is defined by the mean intensity (*M*), and the parasite aggregation parameter *k* (which varies inversely with the degree of parasite aggregation), as displayed in Eq. 1:

1 $$P = 100 \times \left[ {1 - \left\{ {1 + \frac{M}{k}} \right\}^{ - k} } \right]$$

The best-fit negative binomial model to the observed prevalence and intensity (arithmetic) data is presented by the solid line through the data clouds based on a non-linear lest squares fitting technique. The estimated* k*-values for these best-fit functions are printed within each plot in Fig. 5.

There is a clear relationship between the two epidemiological parameters, where the average prevalence changes in a non-linear fashion as the average intensity of infection rises to reach a plateau, as predicted by the negative binomial probability model of the distribution of parasites per host. At low intensities, the prevalence rapidly increases and then plateaus at higher intensities—the level of the plateau varying inversely with the degree of parasite aggregation (small *k*-values, low prevalence, high aggregation; large *k*-values, higher prevalence, less aggregation). This is shown most convincingly for TT infection (Fig. 5b) (*n* = 43). Heterogeneity in the relationship between prevalence and intensity by study is observed for SM (Fig. 5d), which may result from differing patterns of parasite aggregation in different populations due to environmental or social determinants.

### Stool and urine diagnostic techniques

Across the 232 studies analysed, 35% (80/231) used the WHO recommended diagnostic for STH and SM, Kato–Katz, of which only 41% (33/80) specified the slide frequency used. In 15% (12/80) of studies, single Kato–Katz slides were used, which is known to be an unreliable approach, particularly in low-intensity settings [28]. Two slides per sample were used in 23% (18/80) of studies, whilst three, five and eight slides were used in 4% (3/80), 10% (8/80), and 1% (1/80) of studies, respectively. Averaged across all parasites, Kato–Katz performed using one slide gave an average prevalence of 13%, whilst an average prevalence of 10%, 33% and 30%, respectively, was calculated when two, three and five slides were used.

Overall, 19 different diagnostic methods were noted throughout this review**.** Kato–Katz (35%, 80/231) and formal ether concentration technique (FECT) (5%, 34/231) were used predominantly either standalone or in combination (6%, 13/231). Forty-five papers reported the use of multiple diagnostics, which included Kato–Katz, MiniFLOTAC, McMaster, microscopic examination, FECT, Mini Parasep SF faecal parasite counter, wet mount, Baermann technique, PCR, qPCR, point-of-care circulating cathodic antigen test (SM only), Ziehl Neelsen, rapid sedimentation technique, FLOTAC, Schistosomiasis One Step test, and zinc sulphate flotation techniques. The sensitivity of these individual diagnostics varies significantly between methods, which introduces heterogeneity. This lack of diagnostic standardisation compromises the comparability of prevalence, as discussed previously.

### Treatment of STH and SCH

Only 31% (71/231) of the studies noted that any anthelmintic drug (ALB, MBZ, PZQ) was offered to individuals or communities participating in the data collection, with 20% (45/231) vaguely mentioning access to anthelmintics via referral or from the research team. Two papers noted that the treatment cost was passed on to the participants should they wish to be treated. Of the publications identifying the chemotherapeutic offered, 91% studies offered PZQ for SCH and ALB for STH in line with WHO recommendations. MBZ was used for STH treatment in 6% of studies.

Besides the lack of data on the chemotherapeutic used, treatment coverage attained whilst drugs were administered is rarely mentioned. Of the 105 papers detailing treatment, data were only available on surveyed individuals and not for the remaining community. Therapeutic coverage and data on individuals not complying with treatment should be collected to help monitor successes or failures in a control program. Qualitative surveys will help to explain if non-compliance is based on personal choices, or due to a lack of control programme reach through communities.

### Standardisation of reporting

In light of the difficulties faced in generating this review with regard to standardisation, it would be preferable for all future STH and SCH research papers to follow reporting guidelines. This will enable comparability and therefore improve disease prevalence monitoring if analysis of extracted data is optimised. Stratifying prevalence according to age group, which is considered important, will provide important insights into parasite aggregation in the community. Reporting against ubiquitous age bands will further enhance future monitoring and evaluation of control progress, as community pockets of high parasite aggregation can then be identified and targeted. This review did not identify any statistically significant differences in gendered parasite burden; however, the paucity of gender-defined data prevented an in-depth gender-age-stratified analysis.

Suggested criteria for study reporting and age groupings [25, 29] are detailed in Table 2. These guidelines would prevent divergence in reporting parameters and allow in-country and global comparisons.

**Table 2:** Recommended criteria for future SCH and SCH reporting, including age categorisation

Criteria	Definitions
Year(s) of study completion	Year and month—relevance for seasonality in future analyses
No. of participants	Overall and age/sex breakdowns
Sex ratio	Overall and in each age category
Age mid-point	Mean* not* median
Age breakdown	Standardised age boundaries—age range* or* each year of age
Prevalence of parasites	Defined numbers—number infected and/or percentage
Intensity of parasites	Calculated using arithmetic* not* geometric mean
Co-prevalent parasites	Named species found in a single sample
Diagnostic used	Named method and number of replicates (slides and/or days)
Participants treated before	Any exclusion criteria, e.g. anyone treated 1 month prior to study
Treatment offered	Name of drug, dose, days taken. Always to be free to participant
Location of study	GPS coordinates of site(s), or woreda name if privacy concerns dictate
Age groups	Suggested age boundaries
pre-SAC	0–4 Years of age
SAC	5–14 Years of age
Adolescents	14–21 Years of age
Adult	22+ Years of age

*GPS* Global positioning system; for other abbreviations, see Table 1

Table 2 shows the recommended criteria that should be adhered to when reporting any STH or SCH study outcomes to ensure accountability and comparability of future papers. Suggested age categories taken from the WHO guidelines for SAC and the elderly are also given to ensure data reported by future studies are standardised and therefore comparable within age groups and, ultimately, study sites.

## Discussion

The objective of this review was to understand the progress made in moving towards the elimination of STH and SCH in Ethiopia. This review provides epidemiological data on the control of STH and SCH prevalence, and their distribution by region, species, age and diagnostics. This assessment of the current predominantly school-based treatment strategy points toward the need to expand control community-wide.

The overall prevalence of both STH and SCH has decreased in Ethiopia over the last two decades, illustrating the progress made by the national STH and SCH control program in targeting the eradication of these diseases by 2025. The higher number of studies published in the last decade reflect the increased interest in and awareness of NTDs and their vast public health implications. Highest prevalences were recorded for SM and TT, whilst the average prevalence was highest for SM and AL. The high prevalences of AL, SM and TT recorded highlight the importance of improving water, sanitation and hygiene (WaSH) and clean water infrastructure in order to disrupt faecal-oral transmission pathways for STH, and reduce exposure to the infested water bodies that are required for SCH transmission.

Most studies focused on SAC, likely mirroring the focus of Ethiopia’s school-based control programme. There was a lack of publications found on pre-SAC and adults, despite high levels of infection among older individuals documented. Indeed, there were unexpected age-prevalence profiles, with prevalence of AL and TT highest in adults. This may reflect the impact of several rounds of school-based MDA. The high parasite burden in adults will challenge the parasite reduction achieved in children, as this reservoir of infection will likely re-infect SAC, undoing control efforts. Furthermore, the age-prevalence infection pattern of HW was uncharacteristically high among SAC, but remained highest in adults. Untreated adults will act as a reservoir for infection in the community, excreting infective stages that can easily transmit through communities with reduced access to sanitation and clean water.

Increasing the stratification of results by age and gender is crucial for comparative evaluation of national STH and SCH control programmes, and the identification of any pockets of infection within age groups in communities. This stratified information will enable the generation of specific guidelines targeted at specific age groups and/or genders to reduce interaction with infectious material through parasite control, WaSH behaviour change activities and targeted communication materials.

Over the past two decades, SNNPR and Amhara have displayed the highest levels of STH and SCH infection. Nevertheless, SNNPR has consistently displayed a significant reduction in STH and SCH prevalence over the last 20 years. Significant reductions were also seen for some parasites in Amhara, Oromia and Tigray. Areas in southwest and northwest Ethiopia continue to have pockets of higher transmission relative to other areas. These areas may have preferential environmental conditions (temperature and humidity) for parasite transmission, or are geographically remote areas with reduced access to health care and WaSH infrastructure. Geospatial epidemiological studies would be preferential to assess the relationship, if any, between geographical niches and parasite prevalence.

Looking at the spatial location of studies instead of focusing upon political boundaries shows heavy clustering of points in certain regions in central and northern areas. This lack of geographical distribution of studies has reduced any statistical power for comparative statistics, and prevented the scope of analysis to entirely encompass Ethiopia. To our knowledge, no recent publications have reported the geographical locations of studies conducted across Ethiopia, thus reporting these locations in this review will hopefully improve national dispersal as groups aim to work in research-naïve areas. Two regions of particular importance are Gambella and Harari, as they harbour the highest STH and SCH prevalence in the latest national mapping (2013) but have received minimal research attention.

The current national STH and SCH control program targets SAC despite increasing data demonstrating a similar prevalence of AL and TT among pre-SAC and SAC, and significantly higher prevalence of AL, TT, HW, SS and SH in adults. This indicates potential failure if the aim of the national STH and SCH control programme is to achieve elimination. Increasing the treatment age of the groups may see adult prevalence reduce, mirroring the impact clearly made on SAC. Ethiopia should adopt the current WHO recommended strategy to include pre-SAC consistently, women of reproductive age (including those in second, third trimesters, and breast-feeding) and adults in high-risk occupations [8]. To progress further, STH and SCH control could be integrated with the existing infrastructure for current community-wide LF treatment, expanding into STH and SCH endemic areas without LF. This expanded MDA regimen could be strengthened by pairing it with improved waste management, promoted through effective WaSH programmes that ensure mobilisation. High adherence to treatment (> 80%) is imperative to achieving elimination, as persistent non-adherence creates a key reservoir of infection that will prevent transmission break. Once these measures have been implemented, a decrease in the current prevalence levels shown here would be expected to be observed provided that high adherence and compliance are achieved and sustained over several years.

Expanding treatment should decrease the length of time required to treat the community, as modelling studies have predicted SAC exclusive treatment is unlikely to reach transmission break, requiring community-wide treatment to reach this target [30, 31]. Not expanding the treatment protocol raises two questions: will drug resistance develop in these communities under prolonged treatment schedules, and will pharmaceutical donations continue indefinitely?

To monitor and evaluate progress towards elimination, there is a need for the accurate determination of prevalence. This review highlights the heterogeneity in different diagnostic techniques used for STH and SCH as well as their accuracy, particularly in low-intensity settings. As demonstrated in this review, prevalence has dropped nationally with increasing MDA rounds, yet the diagnostics measuring this reduction will have had lowered sensitivity at these low parasite burdens. This phenomenon is of concern for the accurate measurement of transmission elimination.

Considerable debate has surrounded the use of Kato–Katz, the current WHO recommended diagnostic [32]. Kato–Katz sensitivity is dependent upon STH intensity, with single slides reported to be 74–95% (or 62–90% at 300 epg [28]) accurate in high-intensity settings, with accuracy falling to 53–80% (50–80% at 100 epg [28]) in low-intensity settings [33]. The number of slides also plays a role in the accuracy of Kato–Katz, where sensitivity increases with the number of slides used [34]. A third of studies in this review used Kato–Katz, and often used a single slide. As noted by Bärenbold et al [31], single slides in low prevalence settings have a sensitivity of 50%, with sensitivity increasing to 80% with double slides. Therefore, it is recommended that the protocol be changed for Ethiopia, with at least double Kato–Katz slides per sample, or alternatively including molecular techniques where budgets allow. These techniques will improve the diagnosis of SS, which is currently underdiagnosed.

Furthermore, the use of Kato–Katz presents issues for HW and SS diagnosis. The time-lag between slide preparation and reading is crucial for HW as malachite green, which is used to stain stool sample slides, degrades HW eggs. This reduces the window of slide reading to 30-60 min after preparation [35]. SS diagnosis requires alternative techniques to those used for STH. Larvae, rather than eggs, are excreted in the stool and can be seen on faecal slides. The likelihood of the presence and subsequent identification of larvae is small, leading to SS underdiagnosis, as clearly demonstrated in this review. Blood tests are thus preferential, yet unfortunately unrealistic in field settings.

Monitoring MDA treatment efficacy and accurately quantifying infection burden will require diagnostic tools with higher sensitivity, such as PCR and qPCR [36]. In Ethiopia their use is scarce, as reported in 2003 (qPCR) [37], 2013 (PCR) [38], and 2014 (qPCR) [39]. The increased sensitivity achieved through antigen/antibody-based diagnostics makes them preferential for situations where control activity has moved transmission to the end game, and the focus needs to be on the remaining pockets of infection. This is counterbalanced by considerable labour and consumable costs, and unfeasibility in field settings. A high-sensitivity, low-cost diagnostic is still required for ubiquitous use in field settings [15]. In the meantime, egg-counting methods can be improved by using multiple slides over consecutive days. More broadly, WHO must encourage some standardised guidance in the use of alternative diagnostic methods to help facilitate the interpretation of NTD control progress, especially in low prevalence settings.

The non-linear relationship between prevalence and average intensity of infection, whereby prevalence changes negligibly at high intensities and dramatically at lower intensities, raises doubt about the use of prevalence as the standard epidemiological measure. Large changes in mean intensity, which acts to reduce the morbidity burden, are accompanied by very small changes in prevalence [35]. This is crucial knowledge for any study reporting parasite burden, or surveys monitoring the impact of an MDA programme. As a key statistic for defining the WHO morbidity elimination goal, whereby MDA is targeted to reduce moderate and heavy STH infections in pre-SAC and SAC below 2%, and heavy SCH infections to below 1% [23, 24], reporting the arithmetic mean intensity should be specified alongside prevalence by the WHO guidelines.

## Conclusions

STH and SCH are still highly prevalent in some areas of Ethiopia. This review of the literature highlights the heterogeneity in the methods used to assess the prevalence and intensity of helminth infections, in the age groups sampled, regions studied, diagnostic methods used, and lack of data collected that are imperative for control programme evaluation such as treatment coverage. The standardisation of a monitoring and evaluation protocol would facilitate the amalgamation of data for the assessment of progress toward elimination. To reach transmission break (targeted for 2025), achieving STH prevalence of 2% (verified by qPCR) 24 months after MDA programme cessation is required. This 2% target is similarly applied in the 2030 WHO NTD roadmap for STH control, decreasing to 1% for SCH. To achieve this goal, the current school-based national STH and SCH control programme should be expanded to include the whole community as per the current WHO recommended strategy for STH control, including pre-SAC, women of reproductive age, and high-risk adults alongside SAC. An expansion of the age groups targeted by PC in combination with high treatment coverage and other important interventions such as WaSH and behaviour change would increase the likelihood of achieving the ambitious elimination goal by 2025.

## Supplementary Information

**Additional file 1: Figure S1.** Intensity and prevalence age profiles for soil-transmitted helminths (STH).

**Additional file 2: Figure S2.** STH and schistosomiasis (SCH) national mapping data, 2013-2014.

**Additional file 3: Figure S3.** Co-endemicity of STH with SCH and lymphatic filariasis (LF).

**Additional file 4: Figure S4.** Preferred reporting items for systematic reviews and meta-analyses (PRISMA) flowchart of paper selection.

**Additional file 5: Table S1.** Raw data generated from data extraction.

**Additional file 6: Figure S5.** Geographical distribution of studies across Ethiopia.

**Additional file 7: Figure S6.** Change in intensity of STH and SCH between 1994 and 2019.

## Data Availability

All data generated or analysed during this study are included in this published article (and its additional files).

## Abbreviations

AL: *Ascaris lumbricoides*
ALB: Albendazole
epg: Eggs per gram
FECT: Formal ether concentration technique
HW: Hookworm
*k*: Negative binomial parameter
LF: Lymphatic filariasis
MBZ: Mebendazole
MDA: Mass drug administration
NTD: Neglected tropical disease
PC: Preventive chemotherapy
Pre-SAC: Pre-school-age children
PRISMA: Preferred reporting items for systematic reviews and meta-analyses
PZQ: Praziquantel
qPCR: Quantitative polymerase chain reaction
SAC: School-age children
SCH: Schistosomiasis
SH: *Schistosoma haematobium*
SM: *Schistosoma mansoni*
SNNPR: Southern Nations, Nationalities and Peoples’ Region
SS: *Strongyloides stercoralis*
STH: Soil-transmitted helminths
TT: *Trichuris trichiura*
WaSH: Water, sanitation and hygiene
WHO: World Health Organisation
