# Correction: Soil-transmitted helminths and schistosome infections in Ethiopia: a systematic review of progress in their control over the past 20 years

**DOI:** 10.1186/s13071-023-05815-z

**Published:** 2023-06-16

**Authors:** Rosie Maddren, Anna Phillips, Alison Ower, Toby Landeryou, Birhan Mengistu, Ufaysa Anjulo, Ewnetu Firdawek, Nebiyu Negussu, Roy Anderson

**Affiliations:** 1 London Centre for Neglected Tropical Disease Research (LCNTDR), Department of Infectious Disease Epidemiology, School of Public Health, Faculty of Medicine, Imperial College London, London, UK; 2 Children Investment Fund Foundation, London, UK; 3 Disease Prevention and Health Promotion Core Process, Ministry of Health, Wolaita, Ethiopia; 4 Bacterial, Parasitic and Zoonotic Diseases Research Directorate, Ethiopian Public Health Institute, Addis Ababa, Ethiopia; 5 Neglected Tropical Diseases, Federal Ministry of Health, Addis Ababa, Ethiopia

**Correction: Parasites Vectors (2021) 14:97** **https://doi.org/10.1186/s13071-021-04600-0**

Following publication of the original article [1], it was flagged to the journal that the article had published with incorrect affiliation information: all of the authors had been assigned the same affiliation (affiliation ‘1’) in error. The article has now been updated with the correct affiliation information and the correct affiliation information may be found in this erratum. The authors thank you for reading this erratum and apologize for any inconvenience caused.
