# Supplementary material for: Soil-transmitted helminths and schistosome infections in Ethiopia: a systematic review of progress in their control over the past 20 years
Source: Parasit Vectors. 2021 Feb 5;14:97. doi: 10.1186/s13071-021-04600-0 (PMC7866680; doi:10.1186/s13071-021-04600-0)
Supplement: Supplementary file 1 — Additional file 1: Figure S1. Intensity and prevalence age profiles for soil-transmitted helminths (STH). [file 13071_2021_4600_MOESM1_ESM.docx]

### **Fig. S1** Intensity and prevalence age profiles for STH


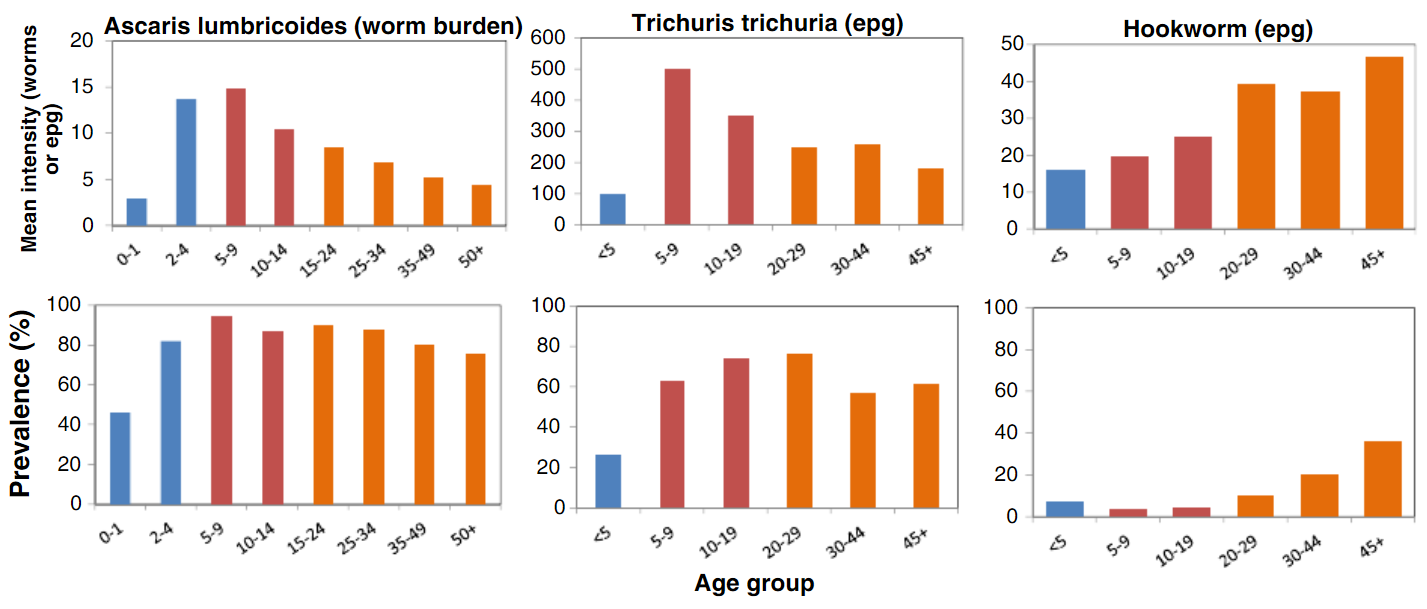


Age-intensity profiles for mean intensity of infection (top row) and prevalence (%, bottom row) for the three major soil-transmitted helminths; AL, TT, and HW. SAC are shown to harbour the heaviest intensity and prevalence burdens with AL and TT infections. This phenomenon is reversed in HW infections, whereby adults harbour higher intensities and prevalence than younger age groups. Taken from Truscott et al, 2014 [9].
