# Supplementary material for: Soil-transmitted helminths and schistosome infections in Ethiopia: a systematic review of progress in their control over the past 20 years
Source: Parasit Vectors. 2021 Feb 5;14:97. doi: 10.1186/s13071-021-04600-0 (PMC7866680; doi:10.1186/s13071-021-04600-0)
Supplement: Supplementary file 2 — Additional file 2: Figure S2. STH and schistosomiasis (SCH) national mapping data, 2013-2014. [file 13071_2021_4600_MOESM2_ESM.docx]

### **Fig. S2** STH and SCH national mapping data, 2013-2014


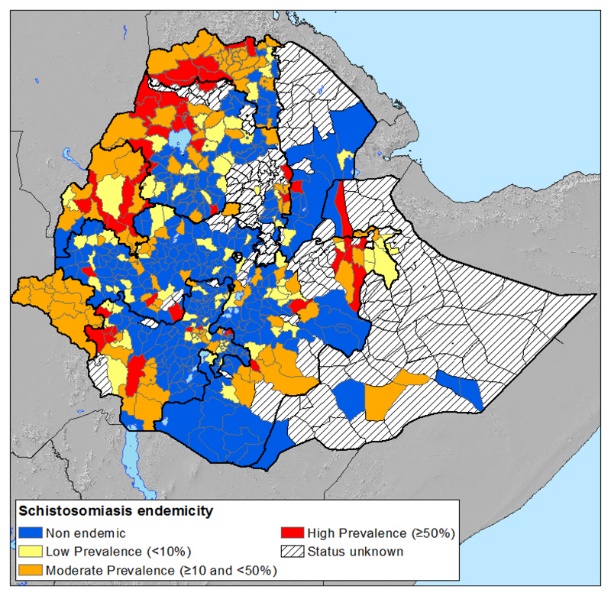

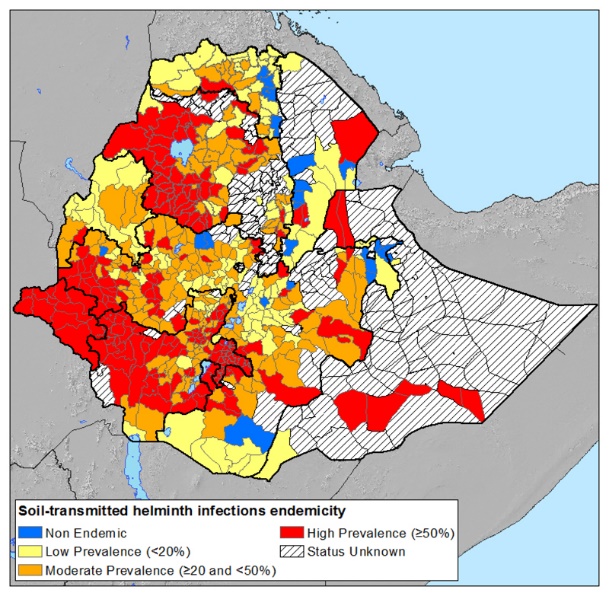


### National Mapping data showing the distribution of STH and SCH categorised according to the WHO guidelines, taken from Negussu et al, 2017[4]. STH and SCH infections are shown at higher prevalence predominantly in south-west and north-westerly regions. Note the large number of regions (depicted as striped grey areas) in which no data is recorded. In part this can reflect both desert areas and districts in which conflict has prohibited data collection (e.g. regions bordering Eritrea, Sudan and Somalia).
