# Supplementary material for: Soil-transmitted helminths and schistosome infections in Ethiopia: a systematic review of progress in their control over the past 20 years
Source: Parasit Vectors. 2021 Feb 5;14:97. doi: 10.1186/s13071-021-04600-0 (PMC7866680; doi:10.1186/s13071-021-04600-0)
Supplement: Supplementary file 3 — Additional file 3: Figure S3. Co-endemicity of STH with SCH and lymphatic filariasis (LF). [file 13071_2021_4600_MOESM3_ESM.docx]

### **Fig. S3** Co-endemicity of STH with SCH and LF


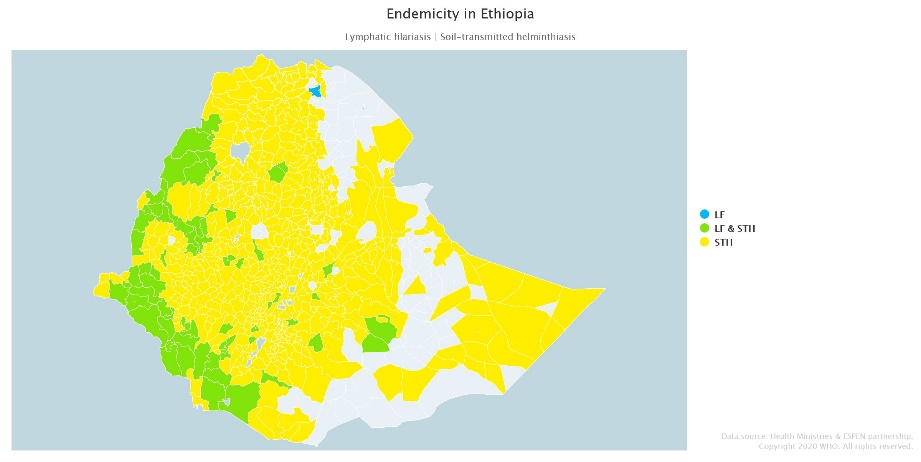

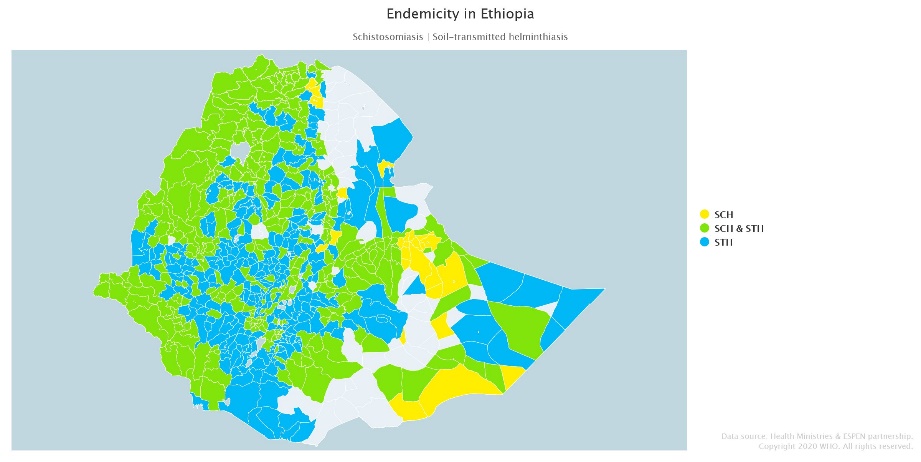

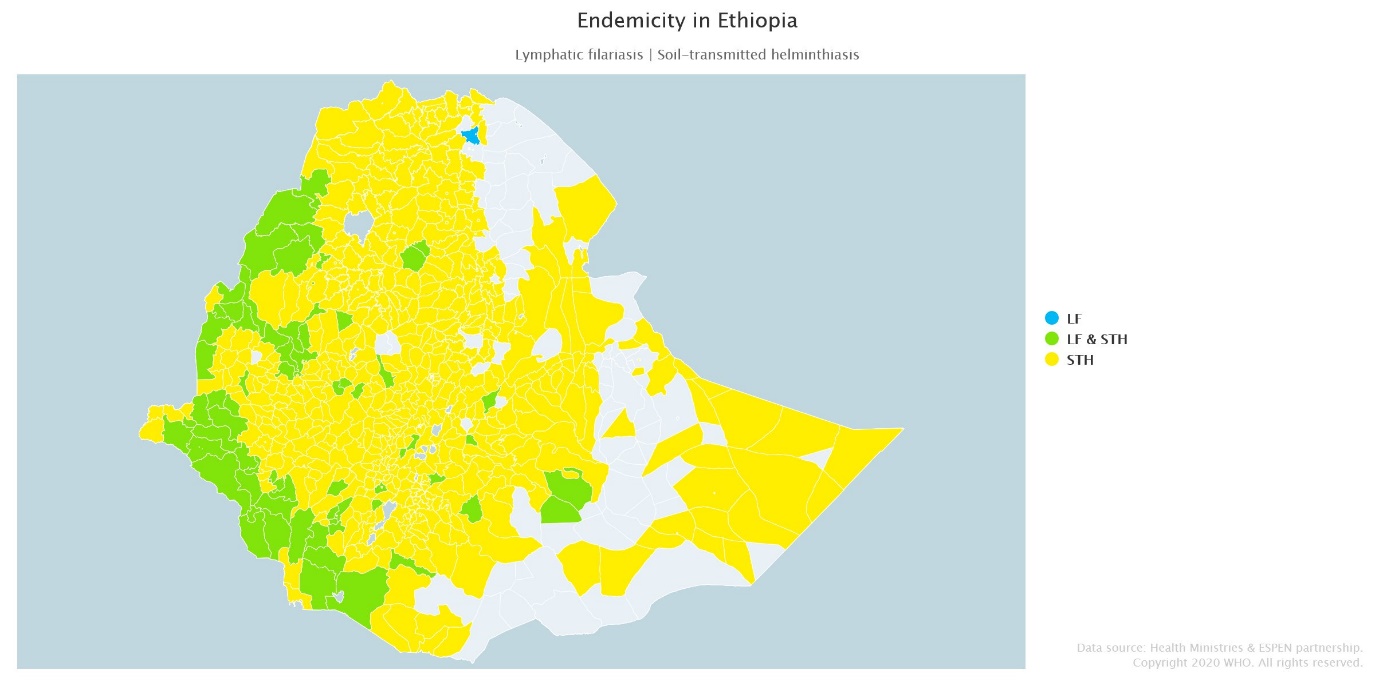

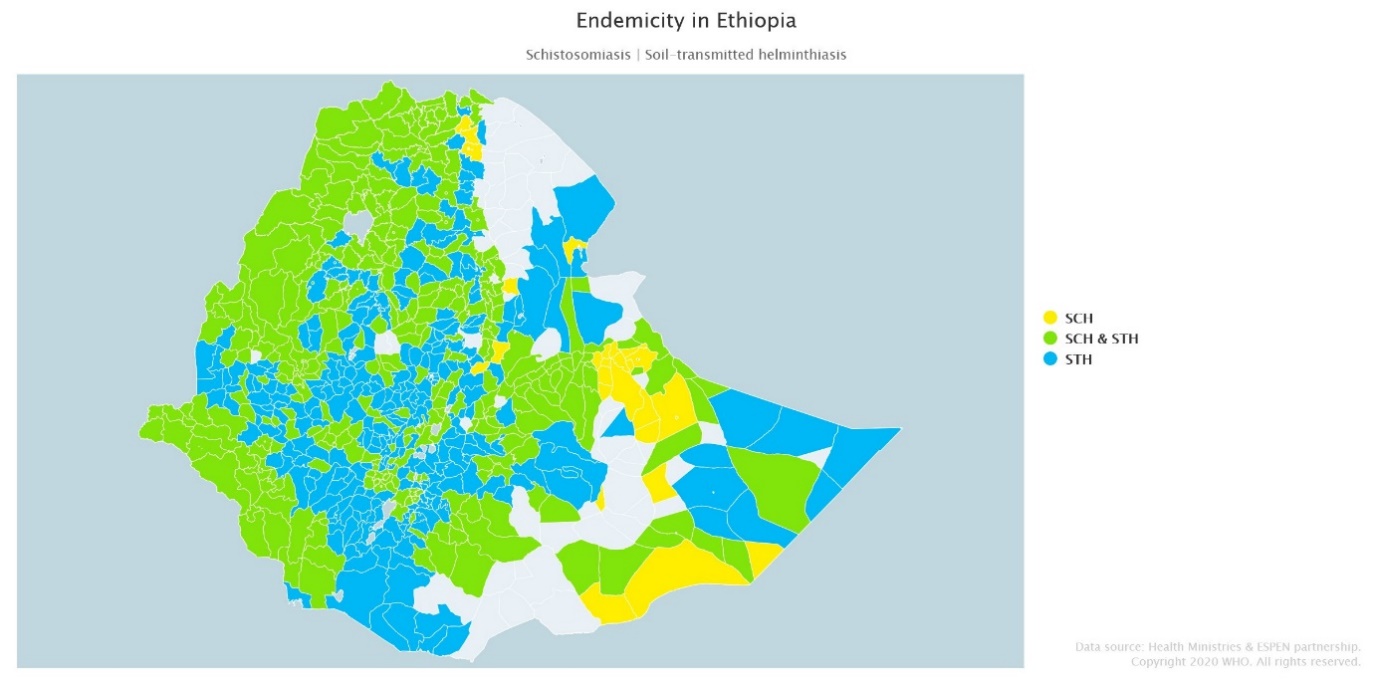


Co-endemicity of STH with LF (left) and SCH (right), demonstrating the heavy co-infection impacting namely western and central regions of Ethiopia. This will have implications not only on individual’s health, but control programme delivery of preventative chemotherapy. Green regions in the LHS map demonstrate LF and STH co-endemic areas, where community-wide treatment with ALB will take place to the benefit of STH control. Data from Health Ministries and ESPEN partnership copyright 2020 WHO [22].
