# Supplementary material for: Soil-transmitted helminths and schistosome infections in Ethiopia: a systematic review of progress in their control over the past 20 years
Source: Parasit Vectors. 2021 Feb 5;14:97. doi: 10.1186/s13071-021-04600-0 (PMC7866680; doi:10.1186/s13071-021-04600-0)
Supplement: Supplementary file 4 — Additional file 4: Figure S4. Preferred reporting items for systematic reviews and meta-analyses (PRISMA) flowchart of paper selection. [file 13071_2021_4600_MOESM4_ESM.docx]

### **Fig. S4** PRISMA flowchart of paper selection


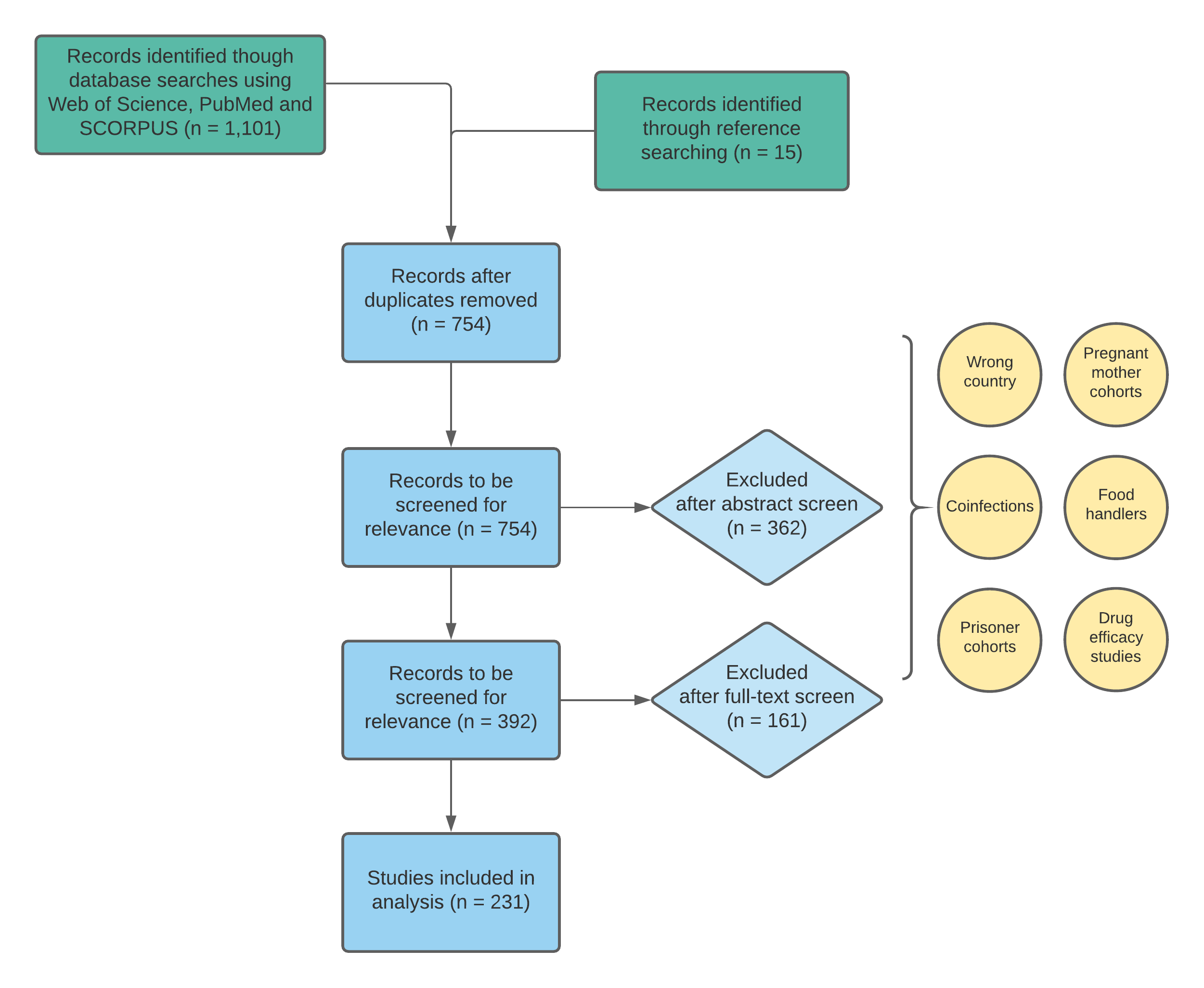


The selection flowchart undertaken for this review, following the PRISMA guidelines. Main themes of the excluded papers are shown in yellow circles. From an initial 754 records, a final 231 records were selected for analysis.
