# Supplementary material for: Soil-transmitted helminths and schistosome infections in Ethiopia: a systematic review of progress in their control over the past 20 years
Source: Parasit Vectors. 2021 Feb 5;14:97. doi: 10.1186/s13071-021-04600-0 (PMC7866680; doi:10.1186/s13071-021-04600-0)
Supplement: Supplementary file 6 — Additional file 6: Figure S5. Geographical distribution of studies across Ethiopia. [file 13071_2021_4600_MOESM6_ESM.docx]

### **Fig. S5** Geographical distribution of studies across Ethiopia

**
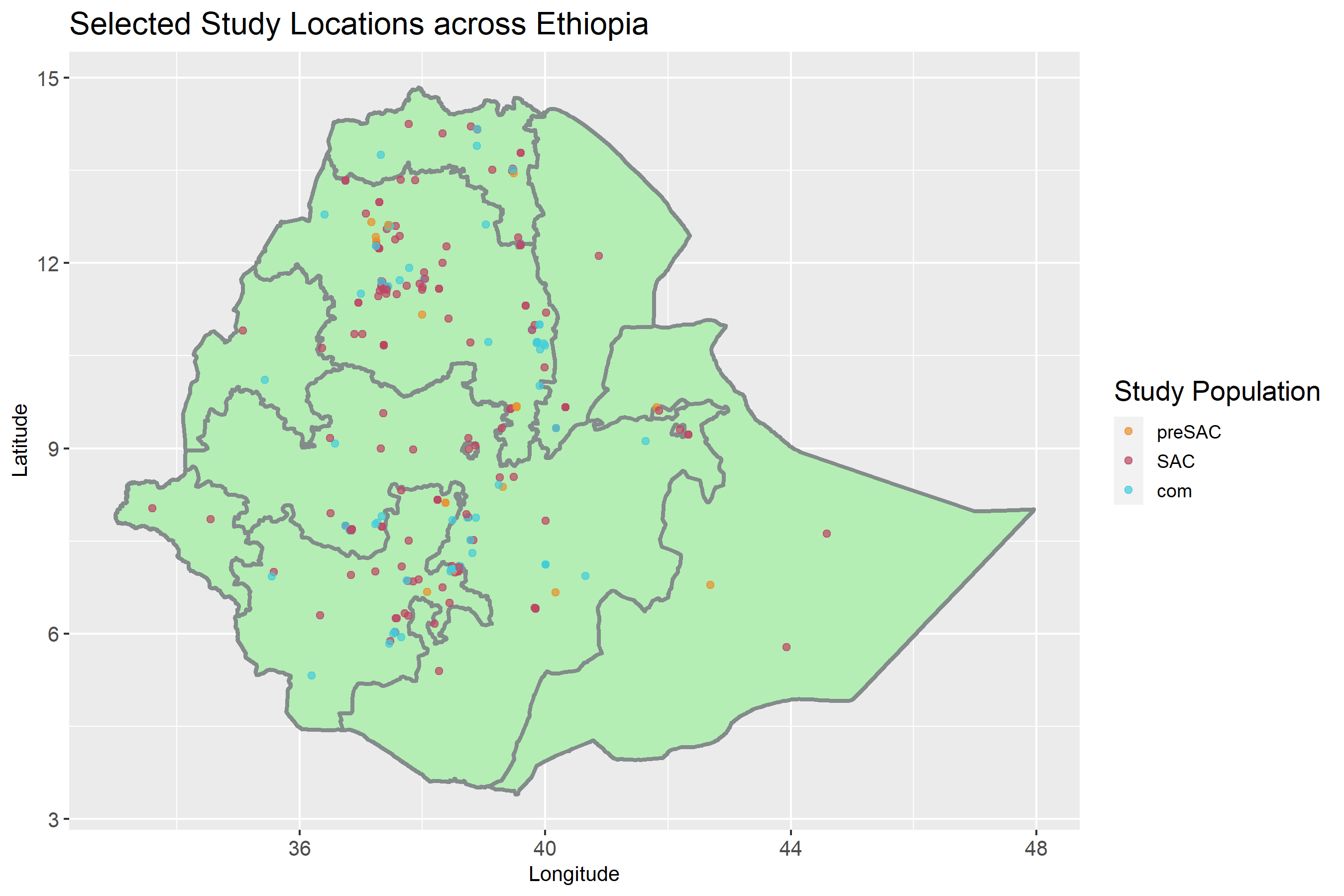
** Locations of the 267 datasets from the 231 selected studies for this review. Clustering of study sites can be seen in Amhara, Oromia and SNNPR. There are limited studies reported from Somali and Tigray, however these arid areas are thought to hinder STH and SCH transmission. Despite western regions such Gambella, Benishangul-Gumuz and westerly SNNPR harbouring the highest prevalence of STH and SCH, there are limited studies conducted in these areas. Study population has been coloured with respect to pre-SAC (orange), SAC (pink), and community-wide(blue) cohorts.
