# Supplementary material for: Soil-transmitted helminths and schistosome infections in Ethiopia: a systematic review of progress in their control over the past 20 years
Source: Parasit Vectors. 2021 Feb 5;14:97. doi: 10.1186/s13071-021-04600-0 (PMC7866680; doi:10.1186/s13071-021-04600-0)
Supplement: Supplementary file 7 — Additional file 7: Figure S6. Change in intensity of STH and SCH between 1994 and 2019. [file 13071_2021_4600_MOESM7_ESM.docx]

### **Fig. S6** Change in intensity of STH and SCH between 1994 and 2019

**
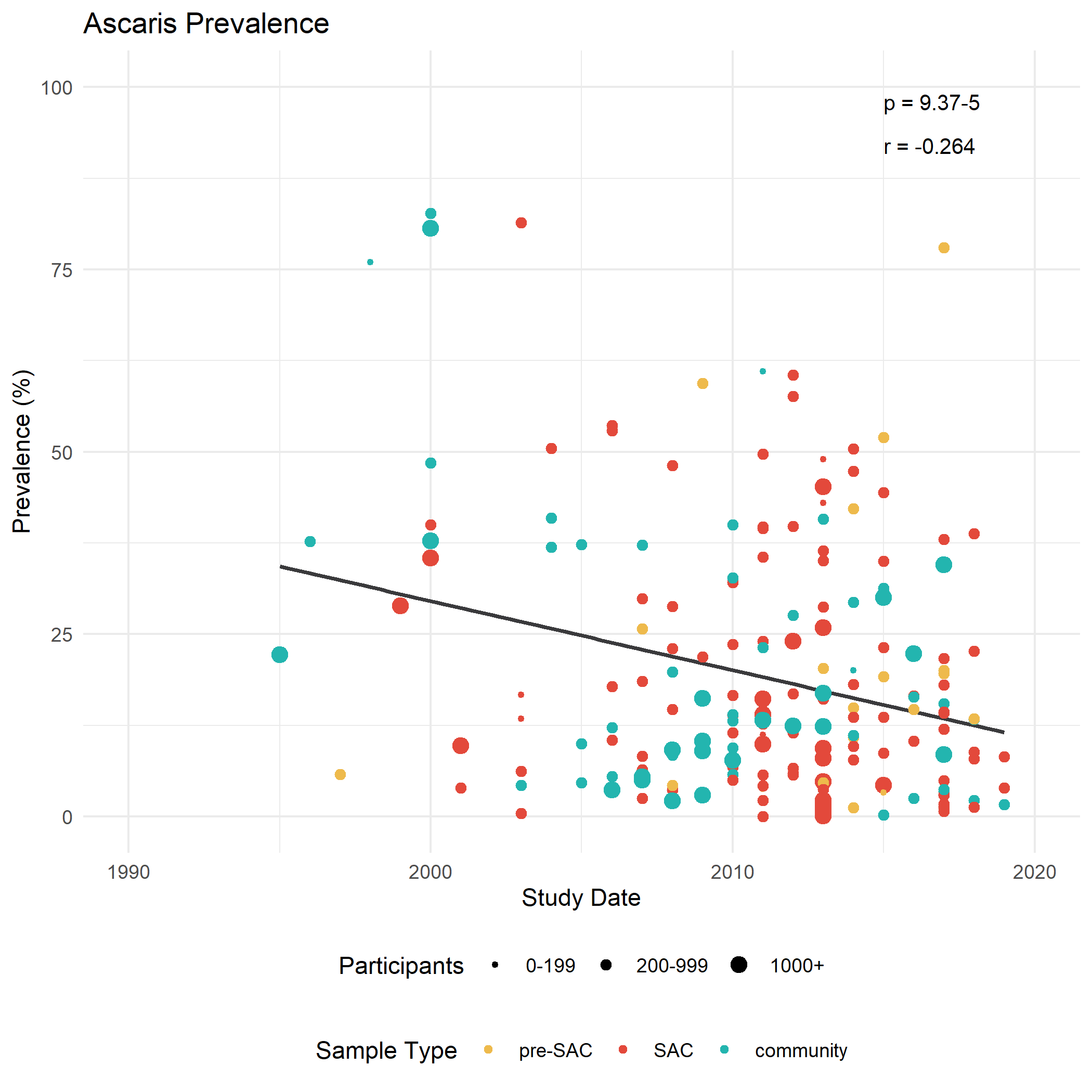
**
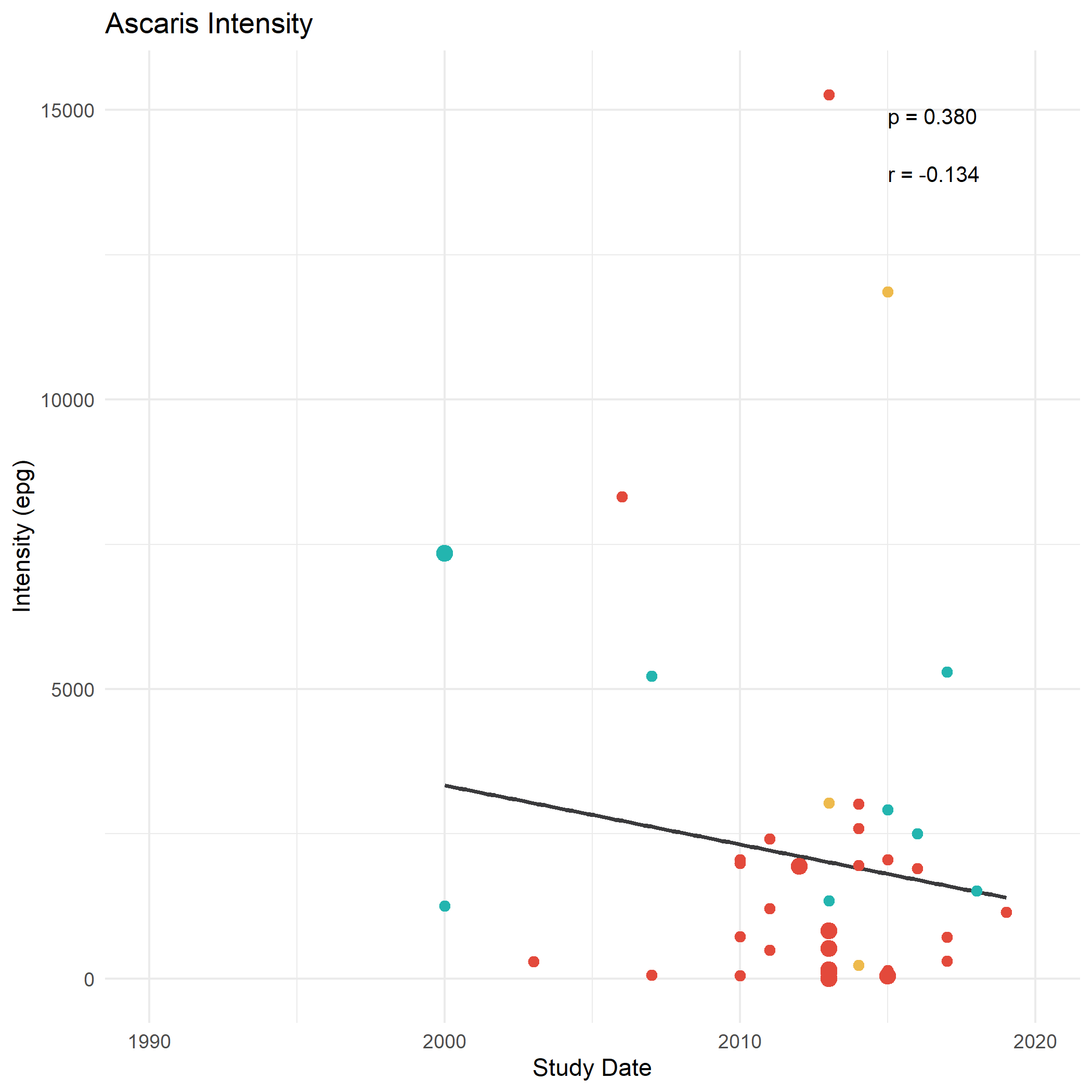

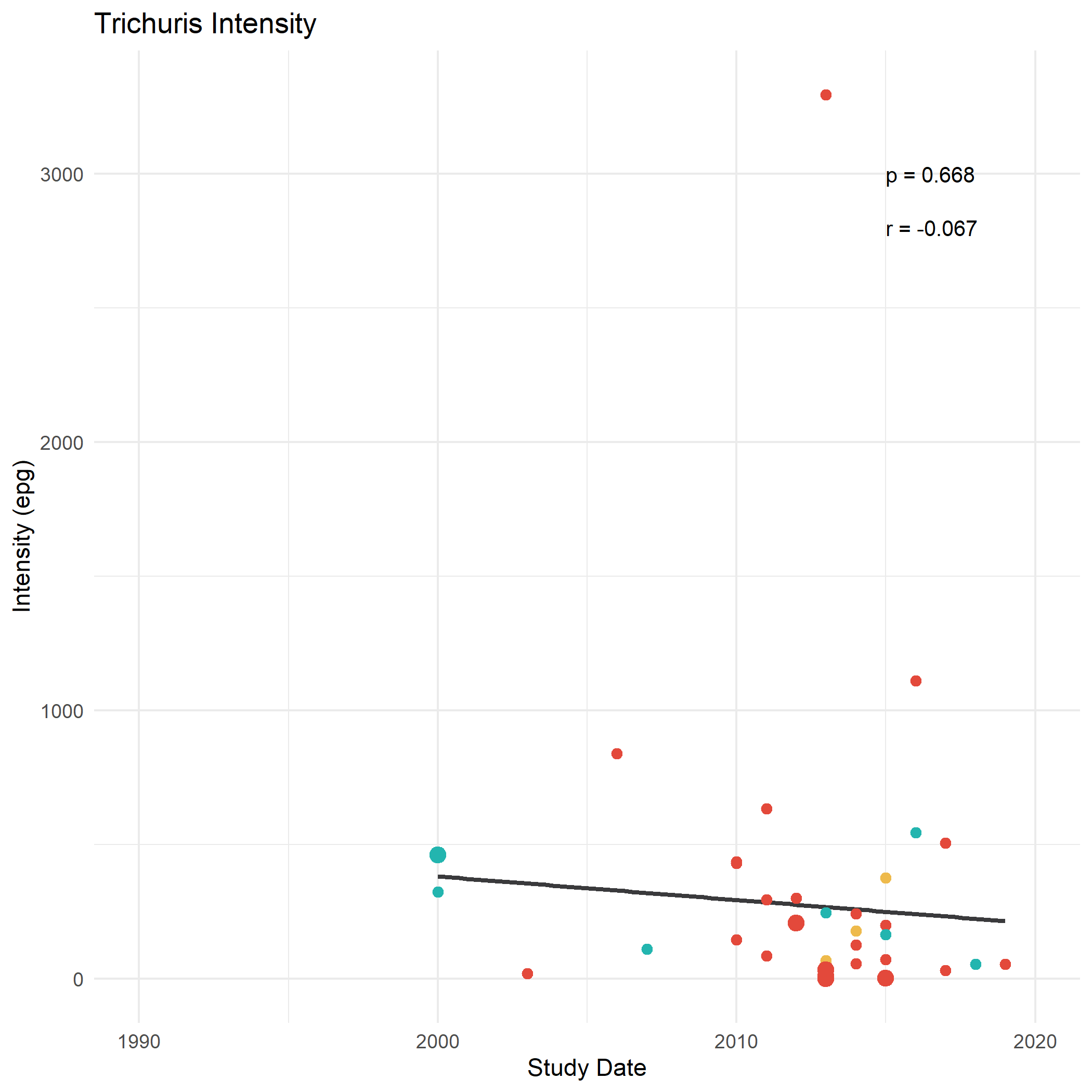

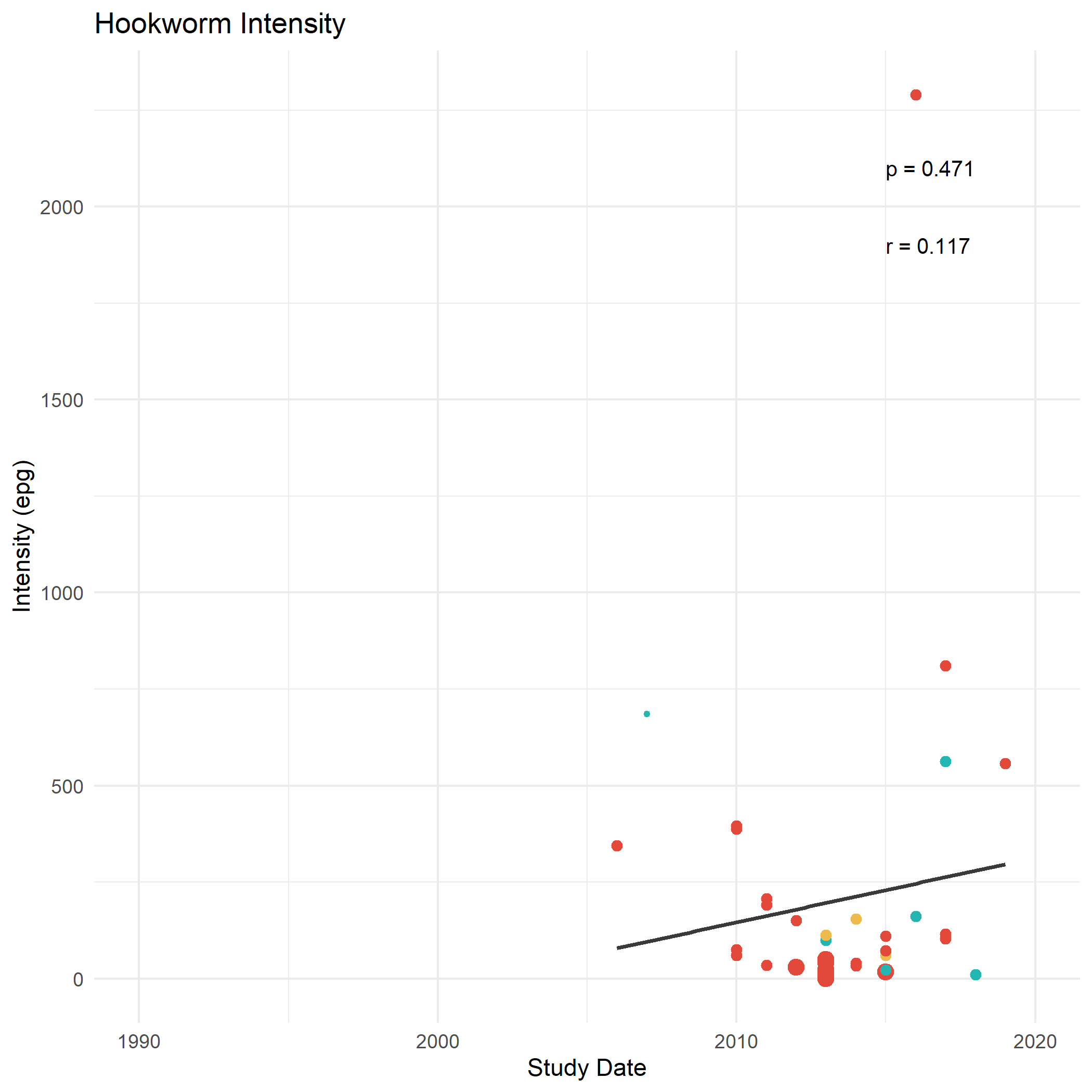

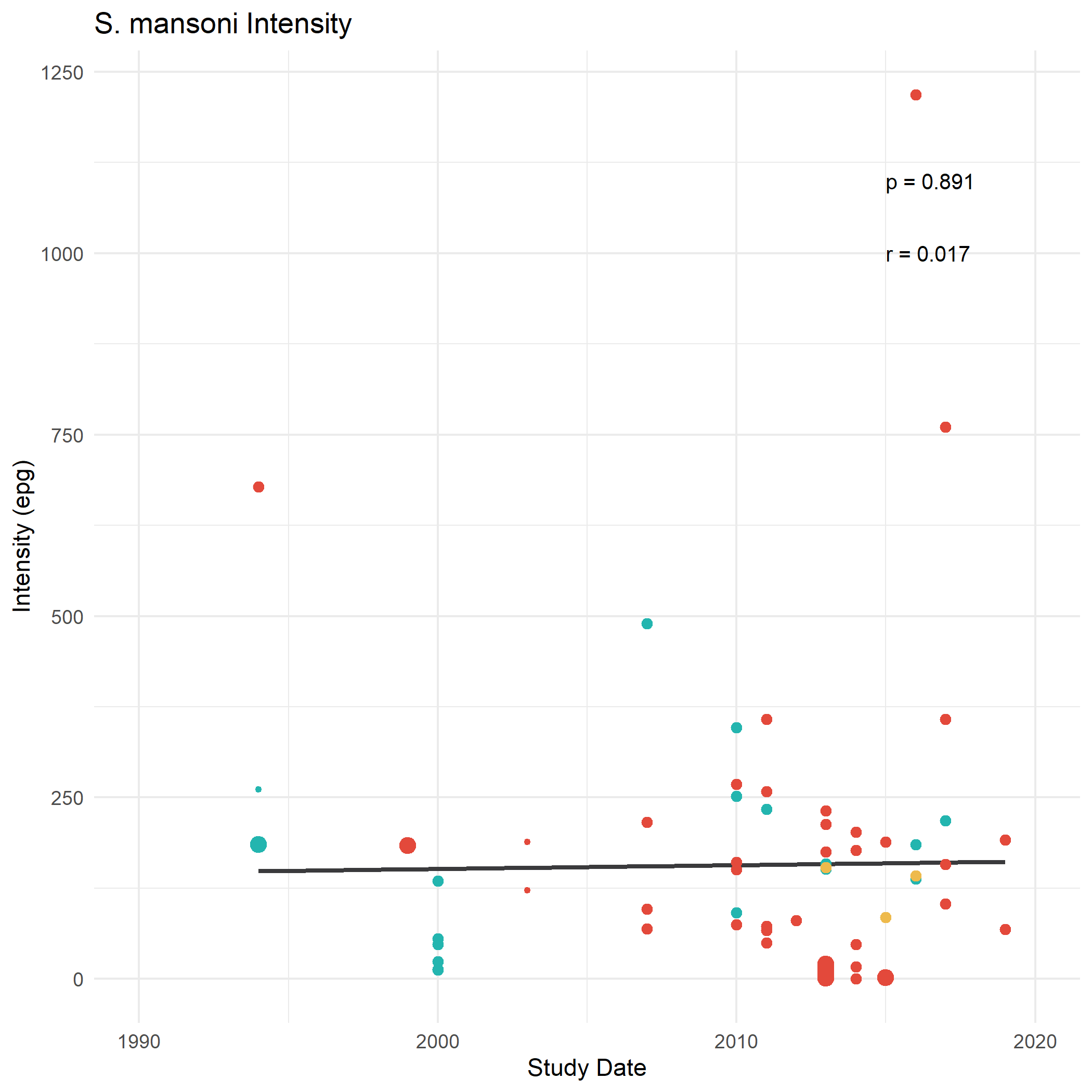


Intensity of STH and SCH infections between 1994 and 2019. Pearson’s correlation coefficient was used to measure the linear correlation between parasite prevalence and study date, printed in the top right corner of each plot with the associated p-value. Study population, differentiating between pre-SAC, SAC and community-wide study populations, has been used to differentiate study data points. The size of points reflects the population size of the studies. The trend line was not weighted by sample size. Note the change in y-axis between parasites.
